# Nutcracker syndrome as the main cause of left renal vein thrombus and pulmonary thromboembolism

**DOI:** 10.1002/iju5.12375

**Published:** 2021-09-20

**Authors:** Kanta Hori, Shota Yamamoto, Maki Kosukegawa, Noboru Yamashita, Yuichiro Shinno

**Affiliations:** ^1^ Department of Urology Otaru General Hospital Otaru Japan; ^2^ Department of Renal and Genitourinary Surgery Hokkaido University Graduate School of Medicine Sapporo Japan; ^3^ Department of Clinical Laboratory Otaru General Hospital Otaru Japan

**Keywords:** left renal vein, nutcracker syndrome, pulmonary embolism, pulmonary thromboembolism, renal vein thrombus

## Abstract

**Introduction:**

Left renal vein thrombus complicating nutcracker syndrome is relatively rare. To the best of our knowledge, there have been only four previous case reports. Furthermore, there have been no reports of pulmonary thromboembolism caused by nutcracker syndrome. Herein, we report a rare case of pulmonary thromboembolism caused by nutcracker syndrome and its clinical management.

**Case presentation:**

A 40‐year‐old man was admitted to our hospital with acute left flank pain. Computed tomography angiography revealed compression of the left renal vein between the aorta and the superior mesenteric artery with a left renal vein thrombus. Furthermore, computed tomography revealed bilateral pulmonary thromboembolism. Rivaroxaban was administered as an anticoagulant. Twenty days after initiation, computed tomography revealed complete resolution of pulmonary thromboembolism and left renal vein thrombus, and repeated computed tomography showed no recurrence.

**Conclusion:**

This case report highlights nutcracker syndrome as a likely cause of pulmonary thromboembolism.

**Abbreviations & Acronyms**
Ao: Aorta
CT: computed tomography
CTA: computed tomography angiography
LRVT: left renal vein thrombus
NCS: nutcracker syndrome
PTE: pulmonary thromboembolism
PV: peak velocity
RVT: renal vein thrombus
SMA: superior mesenteric artery
USG: ultrasonography

**Keynote message**

We report here a case of LRVT and PTE complicating NCS. The most important message of this report is that NCS can cause or significantly contribute to potentially fatal diseases like LRVT and PTE.

## Introduction

NCS refers to compression of the LRV between the aorta and SMA, which results in renal venous hypertension and its clinical manifestations.^1^ The characteristic clinical features of NCS include hematuria, abdominal pain, and left gonadal vein varices. LRVT complicating NCS is relatively rare. To the best of our knowledge, there have been only four case reports of LRVT complicating NCS.^2^, ^3^, ^4^, ^5^

PTE refers to obstruction of the pulmonary vessels by a thrombus, and most cases of PTE are caused by deep vein thrombosis.^6^ PTE is a potentially fatal disease. The overall mortality rate of PTE ranges from 8.1% in stable patients to 25% in those presenting with cardiogenic shock, and 65% in those requiring cardiopulmonary resuscitation.^7^ To the best of our knowledge, there have been no reports of PTE caused by NCS. Herein, we report a case of a 40‐year‐old man presenting with asymptomatic LRVT and PTE complicating NCS. We introduce the case, discuss its clinical features, and summarize the available literature on LRVT associated with NCS.

## Case presentation

A 40‐year‐old man was admitted to our clinic with acute left flank pain. His past medical history included a right ureteral stone and intestinal injury due to a car accident. His vitals were stable. Physical examination revealed isolated left costovertebral angle tenderness. His body mass index was 21.8 kg/m^2^, he was not taking any medications and worked as a truck driver.

Urinalysis revealed microscopic hematuria (5–9/high power field). Laboratory investigations revealed impaired renal function (serum creatinine, 1.13 mg/dL) and raised D‐dimer levels (4.9 μg/mL). Doppler USG revealed compression of the LRV between the abdominal aorta and SMA, as well as an LRVT.

**Fig. 1:**
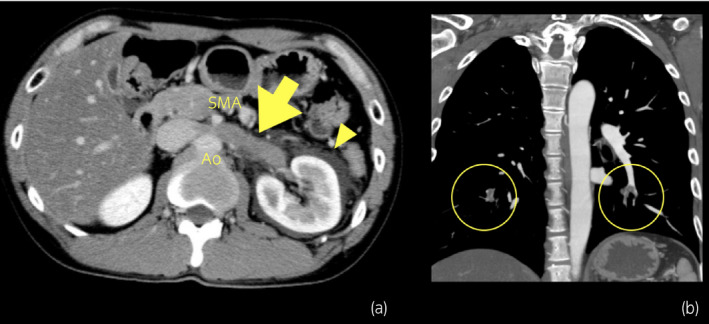
CTA revealed compression of the left renal vein between the aorta and SMA with a left RVT (yellow arrow). Increase in CT‐value around the left kidney indicates congestion by the thrombus (arrow head) (a). CTA revealed bilateral PTE (b).

CTA was performed for better visualization and revealed compression of the LRV between the aorta and the SMA with an LRVT which was found distal to the compression (Fig. 1). Increase in CT‐value around the left kidney indicated congestion caused by the thrombus. Furthermore, CTA revealed bilateral PTE (Fig. 1). There were no findings of deep vein thrombosis or thrombosis other than the LRVT on USG and CTA. Therefore, the pathogenesis of PTE was most likely the LRVT. Laboratory data and imaging studies excluded the presence of other thrombogenic factors such as malignant neoplasm, vascular malformation, trauma, heritable thrombophilia, protein C/S deficiency, and antiphospholipid syndrome. Thus, his only risk factor for venous thromboembolism was his sedentary job as a truck driver.^7^ Based on the above, he was diagnosed with PTE caused by LRVT.

**Fig. 2:**
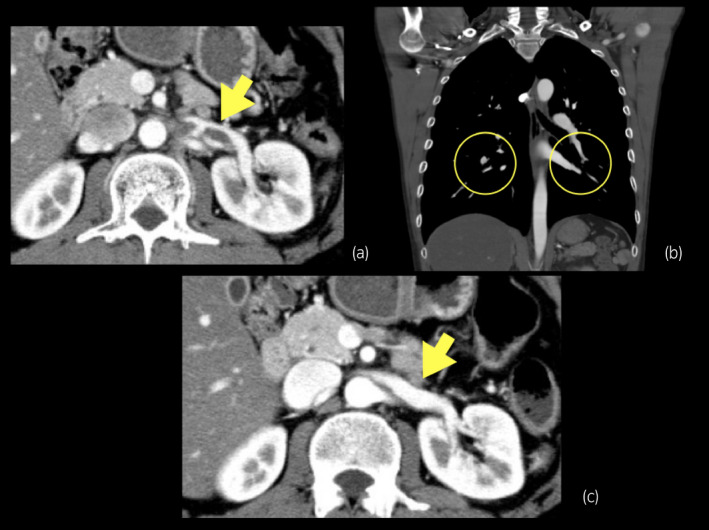
Ten days after the initiation of rivaroxaban, CTA revealed a reduction in the size of the left RVT (a) and disappearance of the PTE (b). Twenty days after initiation of rivaroxaban, CTA revealed complete disappearance of the left RVT (c).

**Fig. 3:**
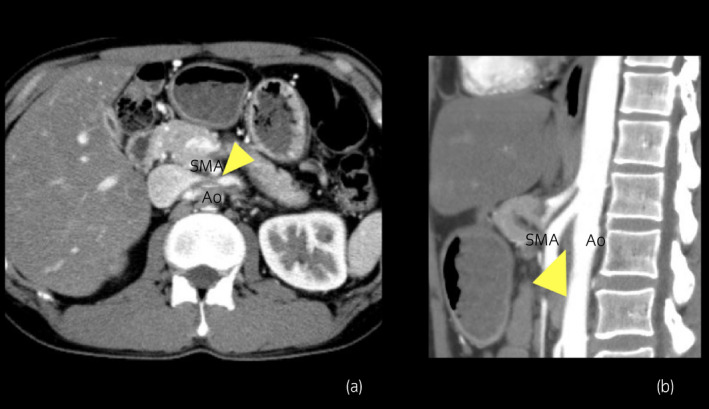
The CTA indicated that the distance between the aorta and SMA at the level of the LRV was 2.5 mm (arrow head) (a). The sagittal CTA showed that the angle between the Ao and SMA was 22° (arrow head) (b).

**Fig. 4:**
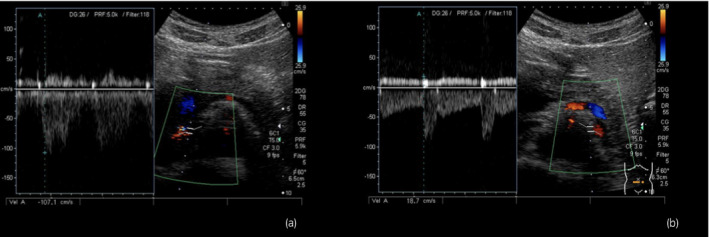
Six months after the initiation of rivaroxaban, USG revealed that the PV at the left renal vein between the aorta and SMA was 107 cm/s (a). The PV at the hilar portion was 18.7 cm/s (b). Using these findings, PV ratio was determined at 5.7.

The patient was reviewed by a cardiology team, and an anticoagulant, rivaroxaban 30 mg daily, was initiated. Since the PTE was mild, an inferior vena cava filter was deemed unnecessary. His left flank pain improved in 3 days. Ten days after the initiation of rivaroxaban, a blood test revealed improvement in renal function (serum creatinine was 0.8 mg/dL) and normalization of D‐dimers (1.0 μg/mL). CTA revealed resolution of PTE and a decrease in LRVT size. Twenty days after initiation, CTA confirmed complete disappearance of the PTE and LRVT (Fig. 2). Thereafter, rivaroxaban was decreased to 20 mg daily. USG and CTA, repeated 3 and 6 months later, showed no recurrence of PTE and LRVT. We used USG and CTA to examine the cause of the LRVT after the PTE and LRVT had completely disappeared. The CTA indicated that the distance between the Ao and SMA at the level of the LRV was 2.5 mm and sagittal CTA showed that the angle between the Ao and SMA was 22° (Fig. 3). We also measured the PV at the hilar portion of the LRV (18.7 cm/s) and at the LRV between the aorta and SMA (107 cm/s) using USG (Fig. 4). The ratio of PV in the LRV between the aorta‐SMA portion and hilar portion was 5.7. Based on the above findings, the patient was diagnosed with NCS, which was also suspected to be the main cause of the LRVT. Rivaroxaban was continued for more than 12 months as PTE carries the risk of recurrence.

## Discussion and conclusion

NCS was first described by De Schepper in 1972.^8^ NCS refers to compression of the LRV between the aorta and SMA, which results in renal venous hypertension and its clinical features. The most common clinical manifestations of NCS include hematuria, abdominal pain, and left gonadal vein varices. LRVT as a complication of NCS is relatively rare. To the best of our knowledge, there have been only four case reports of LRVT complicating NCS.^2^, ^3^, ^4^, ^5^ Furthermore, there have been no reports of PTE caused by NCS. According to the past review of RVT, there are about 30 possible causes for RVT,^9^ the most representative being trauma, renal transplant, volume loss, and hypercoagulability. In our case, the patient’s sedentary job as a truck driver was the only possible cause among those listed in the above review. LRVT has been introduced as complications of NCS in the past articles and guidelines.^10^, ^11^

The diagnosis of NCS requires relatively invasive examinations such as venographic imaging or intra‐arterial digital subtraction angiography. Less invasive examinations such as USG and CTA can be used as alternative modalities for the diagnosis of NCS. Kim *et al*. used USG and measured the PV at the hilar portion of the LRV and at the LRV between the aorta and the SMA.^12^ They found that the cutoff value that may be useful for the diagnosis of NCS is a PV ratio of more than 5.0. Fu *et al*. used CTA and measured the angle between the aorta and the SMA.^13^ The angles were 39.3 ± 4.3° in the NCS group and 90 ± 10° in the control group. They also measured the distance between the SMA and aorta at the level of the LRV. The distances were 12 ± 1.8 mm in the control group and 3.1 ± 0.2 mm in the four patients, respectively. Our case met all these criteria; therefore, the patient was diagnosed with NCS. In line with the four cases of LRVT caused by NCS, our patient was diagnosed with NCS, and the LRVT was found distal to the compression by NSC. This report highlights NCS as a likely cause of PTE.

## Conflict of interest

The authors declare no conflict of interest.

## Approval of the research protocol by an Institutional Reviewer Board

Not applicable.

## Informed consent

The patient provided informed consent for the publication of this manuscript and supplemental images.

## Registry and the Registration No. of the study/trial

Not applicable.
